# Molecular Characteristics of *Carnivore protoparvovirus 1* with High Sequence Similarity between Wild and Domestic Carnivores in Taiwan

**DOI:** 10.3390/pathogens10060671

**Published:** 2021-05-29

**Authors:** Ai-Mei Chang, Chen-Chih Chen

**Affiliations:** 1 International Program in Animal Vaccine Technology, International College, National Pingtung University of Science and Technology, Pingtung 91201, Taiwan; j10685002@g4e.npust.edu.tw; 2 Research Center for Animal Biologics, National Pingtung University of Science and Technology, Pingtung 91201, Taiwan; 3 Institute of Wildlife Conservation, College of Veterinary Medicine, National Pingtung University of Science and Technology, Pingtung 91201, Taiwan; 4 Department of Veterinary Medicine, College of Veterinary Medicine, National Pingtung University of Science and Technology, Pingtung 91201, Taiwan

**Keywords:** *Carnivore protoparvovirus 1*, wild carnivores, domestic carnivore, virus transmission, Taiwan

## Abstract

*Carnivore protoparvovirus 1* (CPPV-1) is a DNA virus causing gastrointestinal disease and immunosuppression in various terrestrial carnivores. Domestic dogs and cats are considered the primary CPPV-1 reservoirs. The habitat overlap of wild carnivores and free-roaming dogs increases the threat of CPPV-1 transmission between them. This study explored the CPPV-1 distribution among wild carnivores in Taiwan through PCR screening and compared the partial capsid protein (VP2) gene sequences from wild and domestic carnivores. In total, 181 samples were collected from 32 masked palm civets (*Paguma larvata*), 63 Chinese ferret badgers (*Melogale moschata*), and 86 crab-eating mongooses (*Herpestes urva*), from 2015 to 2019 were screened for CPPV-1. The average prevalence of CPPV-1 was 17.7% (32/181), with the highest prevalence in masked palm civets (37.5%). In addition, a masked palm civet was coinfected with two CPPV-1 strains. Among the 33 partial VP2 gene sequences, 23 were identical to the sequences amplified from domestic dogs and cats in Asia, and the remaining 10 were identified for the first time. This study supported the circulation of CPPV-1 strains with the same genomic features as domestic carnivores that are also in wild carnivores from the same environment in Taiwan by molecular data. Therefore, further population control and health management of free-roaming domestic carnivores are recommended.

## 1. Introduction

Domestic dogs (*Canis familiaris*) and cats (*Felis catus*) are the most abundant carnivores worldwide [1,2]. They are abundant in human settlements and are considered invasive species in natural environments outside these settlements. An increasing number of domestic dogs and cats can disturb and potentially threaten native fauna through predation, habitat competition, and disease transmission [3]. Considering the close phylogenetic relationship between wild and domestic carnivores, some pathogens infecting domestic carnivores may be transmitted to wild carnivores, causing disease and ultimately population decline in wild carnivores. For instance, rabies virus [4], canine distemper virus (CDV) [5], *Carnivore protoparvovirus 1* (CPPV-1) [6,7], feline immunodeficiency virus (FIV), and feline leukemia virus (FeLV) [8], have been reported to be transmitted between wild and domestic carnivores; this transmission can ultimately affect the population of endemic carnivores. 

CPPV-1 is a highly contagious pathogen belonging to the family *Parvoviridae* [9,10]. Various CPPV-1 variants have been identified, such as feline panleukopenia virus (FPV); canine parvovirus (CPV-2), along with its various antigenic variants CPV-2a, CPV-2b, and CPV-2c [11]; mink enteritis virus (MEV); blue fox parvovirus (BFPV); raccoon parvovirus (RPV); and raccoon dog parvovirus (RDPV) [12]. CPPV-1 is a single-stranded DNA virus, and its gene structure consists of two major open reading frames (ORFs), encoding for nonstructural proteins (NS1 and NS2), and capsid proteins (VP1 and VP2) [13]. 

Domestic carnivores are considered to be the primary reservoirs of CPPV-1 [14]. A survey in South Korea revealed a CPV-2a seroprevalence of 93.8% in the stray dog population [15]. DiGangi et al. [16] screened feral cats for CPPV-1 within 24 h of their arrival at a shelter and identified a feline parvovirus prevalence of 39.8%. Apart from domestic animals, various hosts in the order *Carnivora* have been identified as having CPPV-1 infection [17]. CPPV-1 can infect more than 280 species of Carnivora and can cause a high degree of host strain-specific infection in different carnivores [18,19]. CPV-2 variants have been reported to induce hemorrhagic enteritis, gastroenteritis, and myocarditis in domestic dogs. In addition, CPV-2c has been indicated to induce more severe clinical manifestations than CPV-2a and -2b can [11,20,21]. CPPV-1 infection has been evidenced to reduce the survival rate of wolf (*Canis lupus*) pups [12,13,14]. Creel et al. [22] found that CPPV-1 infection contributes to the high annual mortality rate of African wild dogs (*Lycaon pictus*). Contact with domestic dogs was the primary risk factor for CPPV-1 infection in African wild dogs [14]. 

The Chinese ferret badger (*Melogale moschata*), masked palm civet (*Paguma larvata*), and crab-eating mongoose (*Herpestes urva* ) are endemic carnivores commonly found in rural areas of Taiwan [23]. However, due to human encroachment into their original habitats, an abundance of free-roaming dogs in the rural areas of Taiwan has been recorded [24]. The sympatric distribution of wild and domestic carnivores may increase the risk of pathogen transmission. Therefore, pathogen surveillance of wild carnivores that enter rural areas is necessary for wildlife conservation and disease control planning [25].

CPV-2a, -2b, -2c, and FPV infections have been reported in domestic carnivores and free-roaming leopard cats in Taiwan, with CPV-2c becoming a dominant variant after 2017 [25,26,27]. However, the distribution of CPPV-1 in other wild carnivores in Taiwan remains unclear. The primary objective of this study was to investigate the distribution of CPPV-1 and characterize the present CPPV-1 variants contributing to infection in Taiwanese wild carnivores. We collected samples from animals captured in live traps and from dead individuals to screen for CPPV-1 infection. Molecular screening for CPPV-1 was conducted by a PCR assay targeting the partial VP2 gene region. The positive amplicons were sequenced, and molecular phylogenetic analysis was performed to compare the relationships of amplified CPPV-1 between domestic and wild carnivores. 

## 2. Results

### 2.1. Prevalence of CPPV-1 in the Wild Carnivore Population

Our study was conducted in Taiwan from 2015 to 2019. We collected 118 live-trapped (LT) wild carnivore samples from 6 masked palm civets, 32 Chinese ferret badgers, and 80 crab-eating mongooses. There were 63 found-dead (FD) wild carnivore samples from 26 masked palm civets, 31 Chinese ferret badgers, and 6 crab-eating mongooses (Table 1). The overall prevalence of CPPV-1 in the samples from wild carnivores was 17.7% [32/181, 95% confidence interval (CI): 12.2–23.2%], of which 10.2% accounted for LT individuals (12/118) and 31.7% accounted for FD individuals (20/63). The CPPV-1 prevalence was significantly higher in FD individuals than in LT individuals (chi-squared test; *p* < 0.001).

CPPV-1 infection was detected in all tested species; the highest prevalence was 37.5% [12/32, 95% confidence interval (CI): 20.73–54.27%] of CPPV-1 observed in masked palm civets. The prevalence in adult, juvenile, male, and female wild carnivores was 17.1% (22/129, 95% CI: 10.6–23.5%), 16.7% (6/36, 95% CI: 4.5–28.8%), 21.2% (18/85, 95% CI: 12.5–29.9%), and 15.0% (12/80, 95% CI: 7.2–22.8%), respectively. No significant difference in CPPV-1 detection was found for age (chi-squared test; *p* = 0.844) or sex (chi-squared test; *p* = 0.409; Table 2).

### 2.2. CPPV-1 Variants and Amino Acid Sequence Analysis in Wild Carnivores 

**Figure 1:**
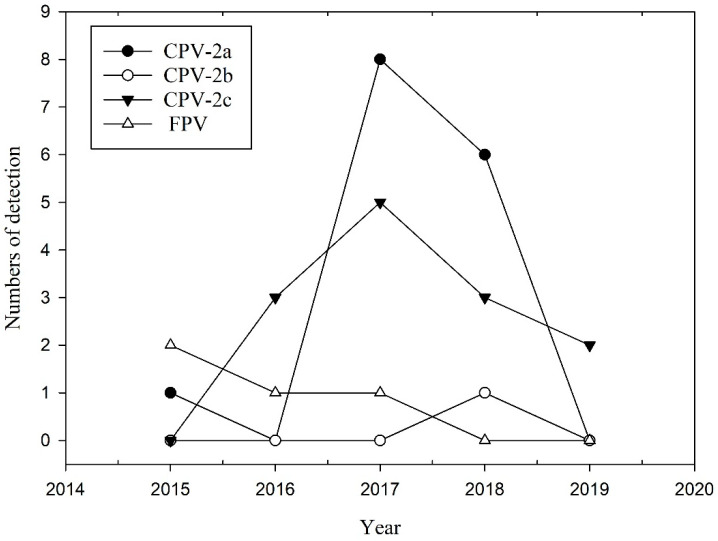
Temporal dynamics of different CPPV-1 variants detected in wild carnivores from 2015 to 2019.

CPPV-1 variants were typed based on amino acid (aa) residues at positions 323, 375, and 426 on the VP2 gene [11,28]. Of the 33 partial VP2 aa sequences amplified from the wild carnivores, 4 were classified as FPV, 15 as CPV-2a, 1 as CPV-2b, and 13 as CPV-2c (Table 2). The temporal dynamic of different CPPV-1 variants obtained in this study is shown in Figure 1. CPV-2a accounted for the majority of the variants detected in the wild carnivore population in this study (Table 2). However, CPV-2a cases tended to decline from 2018 onwards. The CPV-2c cases increased from 2016 onwards. Furthermore, we detected simultaneous infection with two variants, namely CPV-2a and CPV-2c, in one masked palm civet. Based on the comparison of the 33 sequences amplified in this study, the aa sequence types (aaSTs) can be classified into 10 types (Table 3). Most (26/33) of the aaSTs were identical to the predominant sequences of CPPV-1 from domestic carnivores retrieved from the NCBI GenBank, including aaST A, D, E, and H (Table 3). This result indicated that the majority of VP2 sequences obtained from wild carnivores were highly similar to the sequences from domestic carnivores. The aaST B sequence was identical to that obtained from a dog in Pakistan (MF182912); however, the DNA sequence differed from other sequences in Taiwan. Furthermore, we identified five unique aaSTs (C, F, G, I, and J) in our wild carnivores (Table 3). The aaST C showed non-synonymous mutations at Gln309Arg and Gly360Arg from a ferret badger (MT909143); The aaST F showed a non-synonymous mutation at Gln310His from a crab-eating mongoose; The aaST G showed a non-synonymous mutation at Tyr400Asn from a crab-eating mongoose; The aaST I showed a non-synonymous mutation at Glu335Gly from a crab-eating mongoose; The aaST J showed a non-synonymous mutation at Glu411Lys from a ferret badger.

### 2.3. Phylogenetic Analysis of CPPV-1

**Figure 2:**
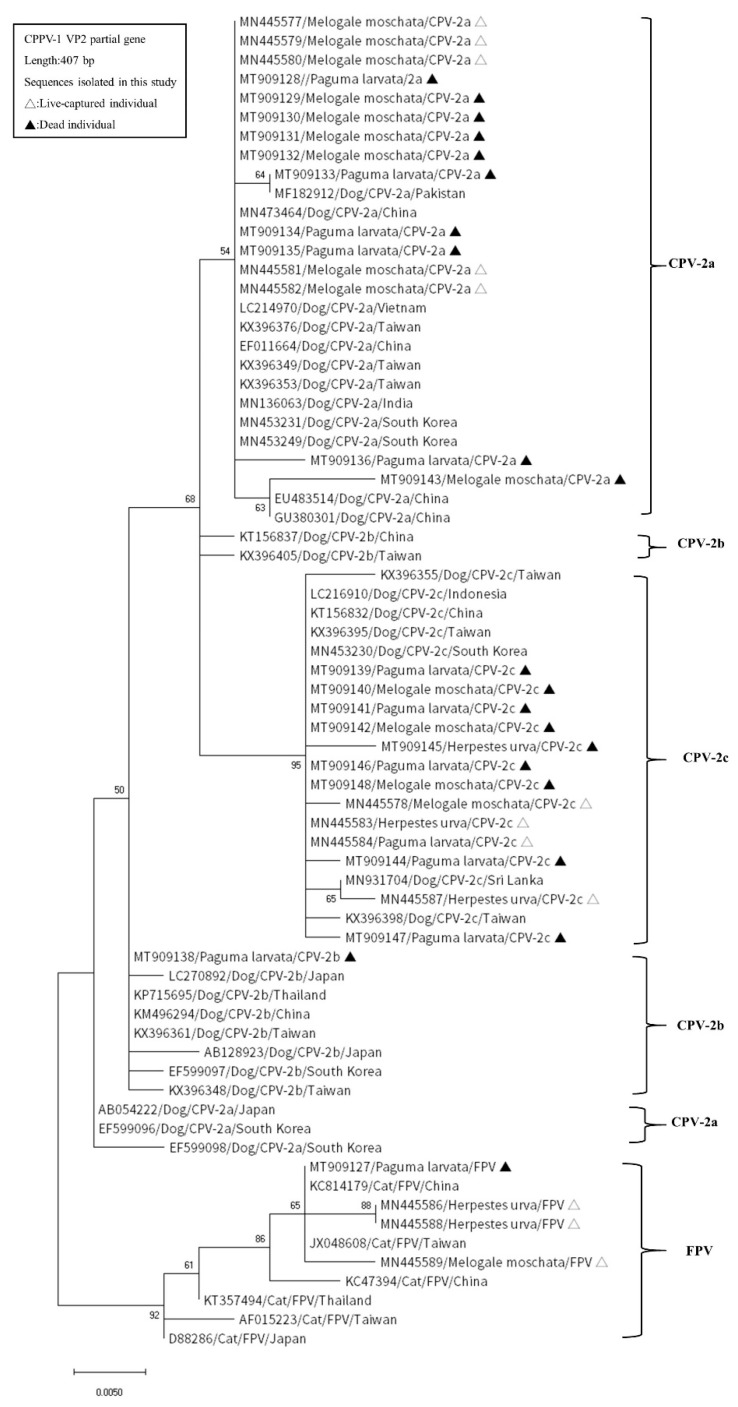
Phylogenetic analysis of partial VP2 nucleotide sequences amplified from the sequences of wild and domestic carnivores in Taiwan obtained from GenBank. Each sequence is labeled with its NCBI accession number, host, viral strain, and country of origin. Sequences from wild carnivores in Taiwan detected in this study are indicated by triangles.

We estimated pairwise distances to show genetic distances in the 10 different aaSTs obtained in this study. The mean genetic distance of CPPV-1 in this study was 0.03 (standard error: 0.01). The distance range between the different aaSTs was 0.00737–0.05389 (Table 4). Phylogenetic tree analysis was conducted based on the nucleotide sequences of the partial VP2 gene. There were 33 sequences amplified from wild carnivores and 37 sequences from dogs and cats retrieved from GenBank that were subjected to phylogenetic analysis. In the phylogenetic tree, the variants CPV-2a, CPV-2b, CPV-2c, and FPV were grouped into distinct clusters based on the variant. In addition, most sequences of each variant amplified from wild and domestic carnivores were identical; therefore, they were distributed in the same subcluster in the phylogenetic tree (Figure 2). 

## 3. Discussion

CPPV-1 has been reported to infect many carnivore species [13,18]. CPPV-1 is generally stable in the environment and can remain infectious for several months. It is transmitted mainly via the fecal–oral route to sympatric carnivores, and the direct or indirect contact with contamination has been demonstrated [29,30]. One of these routes may be scent communication [31]. In specific areas, free-roaming dogs have been indicated as the primary reservoir of CPPV-1, which circulates among the domestic dog population worldwide [32,33]. Furthermore, because of the habitat overlap between domestic and wild carnivores, viral transmission between them is highly possible [34]. For example, Yu et al. [35] reported a novel CPV-2 variant in raccoon dogs and suggested that it might have evolved from a dog in China. In Spain, Olga et al. [36] identified dog- and cat-related sequences in isolates from a wild carnivore. Woodroffe, et al. [14] indicated that dogs were the reservoir host of CPPV-1 transmission to other sympatric wildlife. The results of the phylogenetic analysis in our study supported the likely circulation of CPPV-1 strains among domestic and wild carnivores from the same environments. Following the above findings, dogs and cats could be a probable source of CPPV-1 infection in wild carnivores in Taiwan. 

The first CPPV-1 infection in Taiwan was recorded in 1978 when a high prevalence (99.5%) of stray dogs in animal shelters was reported [37]. In our study, the average prevalence of wild carnivores was 17.7%, and the prevalence of a different sample type, FD individuals (31.7%), was significantly higher than that of LT individuals (10.2%). Similar findings were reported in our leopard cat survey in Maoli, Taiwan [25]. CPPV-1 infection in Asian small-clawed otters and small Indian civets had induced severe clinical signs, including inappetence, lethargy, vomiting, and diarrhea [38,39]. The increased occurrence of vehicle collisions might be attributable to the effect of disease on the behavior and environmental risk awareness of infected animals. An increasing number of road-killed rabbits was recorded in New Zealand from 1994 to 1997, with a high prevalence of rabbit hemorrhagic disease in these road-killed animals [40]. The high prevalence of CPPV-1 infection in FD individuals suggests that this virus could have the potential threat of increasing the occurrence of infected wild carnivores through vehicle collisions in Taiwan.

Research focusing on CPPV-1 in wild carnivores is limited compared with that on domestic carnivores. The first CPPV-1 infection was reported in captive leopard cats (*Prionailurus bengalensis*) and masked palm civets in 1999 based on serological screening [41]. In 2019, the distribution of CPPV-1 in the free-roaming leopard cat population and a comparison of their sequences with those of domestic carnivores were reported [25]. The CPV-2a, CPV-2b, CPV-2c, and FPV variants were found in leopard cats, and the sequences of these CPPV-1 variants were identical to the sequences from domestic carnivores, indicating transmission between leopard cats and domestic carnivores. In the present study, we screened wild carnivores for CPPV-1 infection in the rural area of Taiwan. CPPV-1 infection was found in wild carnivores from three different carnivore families (*Herpestidae*, *Mustelidae*, and *Viverridae*). Moreover, CPPV-1 infection was detected for the first time in Chinese ferret badgers and crab-eating mongooses. CPPV-1 infections by a total of four variants were recorded in wild carnivores in this study. Notably, masked palm civets were infected with all four variants (FPV, CPV-2a, CPV-2b, and CPV-2c). FPV and CPV-2c infection, which causes enteropathy in masked palm civets, was found in Singapore [42]. CPPV-1 transmission among 14 species of sympatric carnivores was also reported in the Serengeti Maasai Mara ecosystem in Tanzania, with a higher prevalence in Viverridae than in *Herpestidae*, *Felidae*, *Canidae*, and *Hyaenidae* [43]. Host susceptibility to CPPV-1 is controlled by host transferrin receptor (TfR). TfR influences the ability of the virus to attach to host cells [44]. The *Viverridae* family is classified in the suborder *Feliformia*, which shares TfR gene similarity with the family *Felidae* [45]. Both FPV and CPV-2 variants can infect domestic cats because of the virus’ ability to bind to feline TfR [46,47]. The sequence of the CPPV-1 gene determines the ability of the virus to infect different hosts [48]. Amino acid residues at positions 93, 300, and 323 of the VP2 gene are located on the surface of the virus, which controls its binding ability [3,12]. Furthermore, residue 305 of the CPV-2 variant had high species specificity. Residue 305 was identified to be Tyr in dogs, but His and Asp in raccoons (Tyr-to-His) and raccoon dogs (Tyr-to-Asp), respectively [12,49]. We did not notice changes in residue 305 in all CPV-2 variants; however, the Asp305Asn mutation of an FPV variant amplified from a Chinese ferret badger (MN445589) was observed.

CPV-2a and -2b have been the major variants circulating in Taiwan for at least two decades. CPV-2c infection in dogs was first reported in 2015 [26,50,51]. CPV-2c has rapidly replaced CPV-2a and -2b as the primary variant circulating in domestic dogs [26]. CPV-2a was the predominant variant in our study, but we also revealed a decrease in CPV-2a infection (Figure 1). CPV-2c infection was first detected in dogs in Taiwan in 2015 [26]. In the present study, we detected CPV-2c infection in 2016. Therefore, the original transmission of CPV-2c might have occurred from domestic carnivores to wild carnivores. The majority of the CPPV-1 sequences amplified from wild carnivores in our study were identical to sequences from the domestic carnivores in Taiwan. Furthermore, an aa substitution, Tyr324Ile, was found in all CPV-2 variants amplified in this study. The first of these aa substitutions have been found in the domestic dog population in Asia [52], including South Korea [53], China [52], Thailand [54], Japan [55], Taiwan [56], and India [57], since 2006. However, through dog importation, this aa changed CPV-2a and 2c has also been reported in Europe since 2014 and since 2017, respectively [58,59,60]. Another substitution, Gln370Arg, was also the most recent of the Asian CPV strains that exhibited change [58,60]. In our study, this substitution change of CPV-2c in wild carnivores was also noticed. The phylogenetic tree placed CPPV-1 from wild carnivores and domestic carnivores in the same subclades. The high similarity of the CPPV-1 VP2 sequences between wild and domestic carnivores indicated the transmission of CPPV-1 between them. Of the unique aaSTs detected in wild carnivores, including aaSTs C, F, G, I, and J, the function of those residues’ mutations was unknown. However, further research on the evolutionary process underlying the adaptation of CPPV-1 to different hosts is required to determine the function of a specific mutation. 

## 4. Materials and Methods

### 4.1. Ethics Statement

Samples from animals were collected in strict accordance with the Wildlife Conservation Act of Taiwan, and permits were obtained from the local administration agencies (National Pingtung University of Science and Technology:1036500723; Kenting National Park:1040005381 and 1050006486; Forestry Bureau, Council of Agriculture;1060002886 and 1070035577). The sampling procedure was approved by the Institutional Animal Care and Use Committee of National Pingtung University of Science and Technology (Approval numbers: NPUST-104-003, NPUST-104-059, NPUST-104-065, NPUST-104-108, NPUST-106-014, NPUST-107-007, and NPUST-109-031).

### 4.2. Sampling Area 

**Figure 3:**
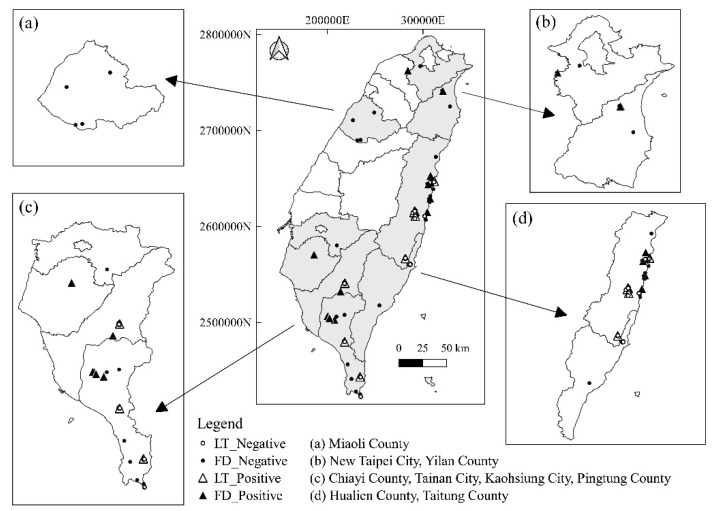
Distribution of sampling areas for wild carnivores in this study, which included Northern, Central, Southern, and Eastern Taiwan. LT, live-trapped; FD, found dead.

The samples of wild carnivores were collected throughout Taiwan, including New Taipei City and Yilan County in Northern Taiwan; Miaoli County in Central Taiwan; Chiayi County, Tainan City, Kaohsiung City, and Pingtung County in Southern Taiwan; and Taitung County and Hualien County in Eastern Taiwan (Figure 3). 

### 4.3. Sample Collection 

The sample collections were from 2015 to 2019. We collected samples from wild carnivores that were live-trapped (LT) or found dead (FD). The target species included the Chinese ferret badger, crab-eating mongoose, and masked palm civet. For the live trapping of small carnivores, we used a metal cage trap (102-Rigid Trap, Tomahawk Live Trap, LLC., Hazelhurst, WI, USA) with chicken liver and heart as bait. The trapped wild carnivores were anesthetized by a veterinarian with a mixture of dexmedetomidine hydrochloride (25 µg/kg) and tiletamine HCl/zolazepam HCl (2 mg/kg). During anesthesia administration, the morphometric parameters included sex and age class (adult, subadult, or juvenile), which were recorded. Rectal swabs and ethylenediaminetetraacetic acid (EDTA)-preserved blood samples were collected for polymerase chain reaction (PCR) screening for CPPV-1. 

Most of the carcasses of carnivores were found dead near human residential areas or roads and deaths were usually caused by vehicle collisions. At necropsy, morphometric parameters which were the same as the live-trapped individuals were collected. Spleen and small intestine tissues, and rectal swabs were collected for CPPV-1 PCR screening.

### 4.4. DNA Extraction, PCR Screening, and CPPV-1 Sequencing 

We extracted DNA from the rectal swab by using a QIAamp DNA fecal mini-kit (Qiagen, Valencia, CA, USA) and EDTA-blood and tissue samples by using a Qiagen DNeasy Blood & Tissue Kit (Qiagen, Valencia, CA, USA), according to the manufacturer instructions. The extracted DNA was subjected to a nested PCR assay for amplification of the partial VP2 gene. Partial VP2 gene amplification was followed by the first PCR amplification of the nested PCR with modified primer from A. Steinel [61], primers M10 (5′-ACA CAY ACA TGG CAA ACA AAT AGA-3′) and M11 (5′-ACT GGT GGT ACA TTA TTT AAT GCA G-3′). In the second PCR amplification, primers M13 (5′-AAA TAG AGC ATT GGG CTTACC ACC ATT TTT-3′) and M14 (5′-ATT CCT GTT TTA CCT CCA ATT GGA TCT GTT-3′) were used. 

The amplification reaction was performed in a 20-μL solution containing 2 μL of 10× PCR Buffer (Mg2^+^plus), 1.6 μL of dNTP mixture (2.5 mM each), 0.2 μM forward and reverse PCR primer, 1.5 U TaKaRa Taq (Takara Shuzo Co. Ltd., Otsu, Japan), and 2 μL of template DNA. The first amplification conditions were as follows: 3 min at 95 °C; 35 cycles of 30 s at 95 °C, 45 s at 52 °C, and 60 s at 72 °C; and a final extension for 10 min at 72 °C. The second amplification of the nested PCR was performed using the same conditions. The expected size of the final nested PCR product was 482 bp. A plasmid containing the VP2 sequence of the commercial CPVV-1 dog vaccine VANGUARD PLUS 5/CV-L (Pfizer Animal Health, New York, NY, USA) was used as the positive control in each assay, and a non-template sample was used as the negative control in each assay to ensure no contamination. PCR amplicons of the expected size were sequenced on an ABI377 sequencer by using the ABI PRISM dye-terminator cycle sequencing ready reaction kit with Amplitaq DNA polymerase (Perkin-Elmer, Applied Biosystems, Foster City, CA, USA). After sequencing, the sequences were edited using Chromas software, version 2.6.5 (Technelysium, South Brisbane, Australia). We compared the sequence similarity using the Basic Local Alignment Search Tool (www.ncbi.nlm.nih.gov/BLAST/ accessed on 27 September 2019) from the National Center for Biotechnology Information (NCBI). All CPPV-1 sequences amplified from wild carnivores in this study have been submitted to the GenBank database. In total, 70 sequences (407 bp) were used for phylogenetic analysis, including 33 sequences amplified from wild carnivores and 37 sequences from domestic dog and cat isolates retrieved from GenBank. The sequences from domestic dogs and cats were obtained using BLAST searches in the nt/nr database of GenBank.

### 4.5. Antigenic Type Characterization and Molecular Phylogenetic Analysis of CPPV-1

Sequences of the partial VP2 gene were used for antigenic type characterization and phylogenetic analysis to compare the sequences between wild and domestic carnivores. The antigen type was classified based on amino acid (aa) residues at positions 323, 375, and 426 on the VP2 gene.

The CPPV-1 DNA sequences were aligned and edited using Clustal W multiple alignment [62] in Molecular Evolutionary Genetics Analysis (MEGA) version X [63]. After alignment, the sequences were trimmed for molecular phylogenetic analysis, in order to prevent the greater length from dominating the analysis. Nucleic acid substitution models with the best fit were identified using the Find Best DNA/Protein Models (ML) in MEGA version X [64]. Ten aaSTs obtained in this study were used for pairwise distance analysis to show the sequence homology. The variations were calculated using a JTT matrix-based model [65]. All ambiguous positions were removed for each sequence pair. There were a total of 135 positions in the final dataset. The number of amino acid substitutions per site from between sequences is shown. Standard error estimate(s) are shown above the diagonal and were obtained by a bootstrap procedure (5000 replicates).

For phylogenetic tree analysis, maximum likelihood estimation was applied based on the Tamura 3-parameter model with the lowest Bayesian information criterion to analyze the phylogenetic relationship among different strains amplified from domestic and wild carnivores [66]. Bootstrapping with 5000 replications was conducted to assess the statistical confidence level in the branching order of the phylogenetic tree [64]. 

### 4.6. Genbank Sequence Submission

Nucleotide sequence of CPPV-1 listed belowed were colected in this study and summited to Genbank, Ferret-badger: MN445577, MN445579, MN445580, MN445581, MN445582, MT909129, MT909130, MT909131, MT909132, MT909143, MN445578, MT909140, MT909142, MT909148, MN445589; Formosan gem-faced civet: MT909128, MT909134, MT909135, MT909133, MT909136, MT909138, MN445584, MT909139, MT909141, MT909144, MT909146, MT909147, MT909127; Crab-Eating Mongoose: MN445583, MN445587, MT909145, MN445586, MN445588. The detail of each individual information was provided in Supplementary Table S1.

### 4.7. Statistical Analyses

The data is expressed at a 95% confidence interval. The prevalence between different sample types (LT and FD), hosts, sex, and age classes were evaluated using the chi-squared test. To estimate the prevalence, the software Sigma Plot Version 14.0 (Systat Software, San Jose, CA, USA) is used in this research. A difference is considered significant at *p* < 0.05. 

## 5. Conclusions

Our study reported the circulation of CPPV-1 in the masked palm civet, Chinese ferret badger, and crab-eating mongoose in rural Taiwan. The detection of CPPV-1 in wild carnivores indicated that CPPV-1 infection is widespread in sympatric carnivores in Taiwan, with CPV-2a being the most prevalent variant and CPV-2c infection increasing in prevalence. The high similarity of the CPPV-1 sequences indicated that the same virus strains were circulated in Taiwan and affected both domestic and wild carnivores in the same period. In addition, the majority of amplicons (32/33) had the same host-specific 305 aa residue. Considering the high density of free-roaming domestic carnivores, CPPV-1 infection may have been transmitted from domestic to wild carnivores in Taiwan. Therefore, further population control and health management of free-roaming domestic carnivores are necessary for preventing pathogen transmission and protecting wild carnivores.

## Figures and Tables

**Table 1:** Species, age, and sex of wild carnivores screened for *Carnivore protoparvovirus 1* in Taiwan, 2015–2019.

Family, Species	Common Name	Sample Type	Age				Sex			Total
			Adult	Subadult	Juvenile	ND ^1^	Female	Male	ND ^1^	
Herpestidae, *Herpestes urva*	Crab-eating mongoose	LT ^2^	53	25	0	2	43	36	1	80
		FD ^3^	5	0	1	0	1	2	1	6
Mustelidae, *Melogale moschata*	Chinese ferret badger	LT	25	5	0	2	11	18	3	32
		FD	29	1	0	1	15	16	0	31
Viverridae, *Paguma larvata*	Masked palm civet	LT	4	1	0	1	2	3	1	6
		FD	13	4	0	9	8	10	8	26

^1^ ND = no data. ^2^ LT = live trapped. ^3^ FD = found dead.

**Table 2:** Prevalence and variants of *Carnivore protoparvovirus* 1 infection in wild carnivore samples.

Species	Sample Type	No. Individuals	No. Positive	Prevalence	95% CI		Variants			
					Lower	Upper	FPV	CPV-2a	CPV-2b	CPV-2c
Crab-eating mongoose	LT ^1^	80	4	5.0%	0.2%	9.8%	2	0	0	2
	FD ^2^	6	1	16.7%	0.0%	46.5%	0	0	0	1
	Sum	86	5	5.8%	0.9%	10.8%				
Masked palm civet	LT	6	1	16.7%	0.0%	46.5%	0	0	0	1
	FD	26	11 ^3^	42.3%	23.3%	61.3%	1	5	1	5
	Sum	32	12	37.5%	20.7%	54.3%				
Chinese ferret badger	LT	32	7	21.9%	0.2%	9.8%	1	4	0	1
	FD	31	8	25.8%	10.4%	41.2%	0	5	0	3
	Sum	63	15	23.8%	13.3%	34.3%				
Total		181	32	17.7%	12.1%	23.2%	4	15	1	13

^1^ LT = live trapped. ^2^ FD = found dead. ^3^ Two virus subtypes, namely CPV-2a and CPV-2c, were detected in one masked palm civet.

**Table 3:** Nucleotide and amino acid (aa) variation in partial VP2 gene amplified from wild carnivores. According to **aa** positions 323 and 426, amino acid sequence types (aaSTs) A to C were categorized as CPV-2a, aaST D was categorized as CPV-2b, aaST E–G were categorized as CPV-2c, and aaST H–J were categorized as FPV. Non-synonymous mutations are bolded.

aaSTs ^1^	Viral Variant	*n* ^2^	aa Position (Nucleotide Position)												
			303 (907–909)	305 (913–915)	308 (923–924)	309 (925–927)	310 (928–930)	323 (967–969)	335 (1003–1005)	360 (1078–1080)	373 (1117–1119)	400 (1198–1200)	411 (1231–1233)	426 (1276–1278)	429 (1285–1287)
A	CPV-2a	12	Phe (TTT)	Tyr (TAT)	Val (GTT)	Gln (CAA)	Gln (CAA)	Asn (AAC)	Glu (GAG)	Gly (GGA)	Asp (GAT)	Tyr (TAT)	Glu (GAA)	Asn (AAT)	Val (GTA)
B	CPV-2a	1	Phe (TTT)	Tyr (TAT)	Val (GTT)	Gln (CAA)	Gln (CAA)	Asn (AAC)	Glu (GAG)	Gly (GGA)	**Gly** **(GGT)**	Tyr (TAT)	Glu (GAA)	Asn (AAT)	Val (GTA)
C	CPV-2a	1	Phe (TTT)	Tyr (TAT)	Val (GTT)	**Arg** **(CGA)**	Gln (CAA)	Asn (AAC)	Glu (GAG)	**Arg** **(AGA)**	Asp (GAT)	Tyr (TAT)	Glu (GAA)	Asn (AAT)	Val (GTA)
D	CPV-2b	1	Phe (TTT)	Tyr (TAT)	Val (GTT)	Gln (CAA)	Gln (CAA)	Asn (AAC)	Glu (GAG)	Gly (GGA)	Asp (GAT)	Tyr (TAT)	Glu (GAA)	**Asp** **(GAT)**	Val (GTA)
E	CPV-2c	11	Phe (TTT)	Tyr (TAT)	Val (GTT)	Gln (CAA)	Gln (CAA)	Asn (AAC)	Glu (GAG)	Gly (GGA)	Asp (GAT)	Tyr (TAT)	Glu (GAA)	**Glu** **(GAA)**	Val (GTA)
F	CPV-2c	1	Phe (TTT)	Tyr (TAT)	Val (GTT)	Gln (CAA)	**His** **(CAT)**	Asn (AAC)	Glu (GAG)	Gly (GGA)	Asp (GAT)	Tyr (TAT)	Glu (GAA)	**Glu** **(GAA)**	Val (GTA)
G	CPV-2c	1	Phe (TTT)	Tyr (TAT)	Val (GTT)	Gln (CAA)	Gln (CAA)	Asn (AAC)	Glu (GAG)	Gly (GGA)	Asp (GAT)	**Asn** **(AAT)**	Glu (GAA)	**Glu** **(GAA)**	Val (GTA)
H	FPV	2	Phe (TTT)	**Asp** **(GAT)**	Val (GTT)	Gln (CAA)	Gln (CAA)	**Asp** (GAC)	Glu (GAG)	Gly (GGA)	Asp (GAT)	Tyr (TAT)	Glu (GAA)	Asn (AAT)	Val (GTA)
I	FPV	1	Phe (TTT)	**Asp** **(AAT)**	Val (GTT)	Gln (CAA)	Gln (CAA)	**Asp** (GAC)	**Gly** **(GGG)**	Gly (GGA)	Asp (GAT)	Tyr (TAT)	Glu (GAA)	Asn (AAT)	Val (GTA)
J	FPV	1	Phe (TTT)	**Asn** **(GAT)**	Val (GTT)	Gln (CAA)	Gln (CAA)	**Asp** (GAC)	Glu (GAG)	Gly (GGA)	Asp (GAT)	Tyr (TAT)	Lys (AAA)	Asn (AAT)	Val (GTA)

^1^ aaSTs = amino acid sequence types. ^2^ *n* = number of partial CPPV-1 sequences amplified from wild carnivores with the same amino acid sequence types.

**Table 4:** Phylogenetic distance matrix presenting amino acid pairwise distances, calculated using MEGA X, among the partial VP2 amino acid sequence of CPPV-1. There was a total of 135 positions in the analysis, and the overall mean (SE) of genetic distance was 0.03 (0.01).

aaSTs ^1^	Viral Variant	aaSTs									
		A	B	C	D	E	F	G	H	I	J
A	CPV-2a										
B	CPV-2a	0.00738									
C	CPV-2a	0.01474	0.02224								
D	CPV-2b	0.01485	0.02241	0.02985							
E	CPV-2c	0.02263	0.03035	0.03789	0.02997						
F	CPV-2c	0.02258	0.03027	0.03780	0.02989	0.01480					
G	CPV-2c	0.01503	0.02267	0.03019	0.02238	0.00738	0.00737				
H	FPV	0.02235	0.02997	0.03743	0.02231	0.04595	0.04584	0.03814			
I	FPV	0.02995	0.03766	0.04515	0.02990	0.05389	0.05376	0.04601	0.00740		
J	FPV	0.02993	0.03762	0.04511	0.02987	0.05384	0.05370	0.04596	0.01479	0.02230	

^1^ aaSTs = amino acid sequence types.

## Data Availability

The data presented in this study are contained within the article and supplementary materials.

## Supplementary Materials

The following are available online at https://www.mdpi.com/article/10.3390/pathogens10060671/s1, Table S1: Characteristics of each CPPV-1-positive individual of GenBank accession, Sample ID, species, age (Ad = adult, Juv = Juvenile), sex (M = male, F = female), sample type and subtype of virus.
