# Supplementary material for: Molecular Characteristics of Carnivore protoparvovirus 1 with High Sequence Similarity between Wild and Domestic Carnivores in Taiwan
Source: Pathogens. 2021 May 29;10(6):671. doi: 10.3390/pathogens10060671 (PMC8229444; doi:10.3390/pathogens10060671)
Supplement: Supplementary file 1 [file pathogens-10-00671-s001.zip › pathogens-1209875-supplementary.pdf]

Table S1. Characteristics of each CPPV-1-positive individual of GenBank accession, Sample ID, species, age (Ad = adult, Juv = Juvenile), sex (M = male, F = female), sample type and subtype of virus.

GenBank Accession	Sample ID	Species	Age	Sex	Sample Type	Antigenic Types
MN445577	2018081501	Ferret-badger	Juv	F	EDTA	CPV-2a
MN445579	2017102101	Ferret-badger	Ad	M	EDTA	CPV-2a
MN445580	2017100101	Ferret-badger	Ad	M	EDTA	CPV-2a
MN445581	2017093002	Ferret-badger	Ad	M	EDTA	CPV-2a
MN445582	2017093001	Ferret-badger	Ad	M	EDTA	CPV-2a
MT909128	C2015102101	Formosan gem-faced civet	U[1]	U[1]	Small intestine	CPV-2a
MT909129	C2017092701	Ferret-badger	Ad	F	Spleen	CPV-2a
MT909130	C2017100901	Ferret-badger	Juv	M	Spleen	CPV-2a
MT909131	C2017102201	Ferret-badger	Ad	M	Small intestine	CPV-2a
MT909132	C2018020801	Ferret-badger	Ad	M	Small intestine	CPV-2a
MT909134	C2018060601	Formosan gem-faced civet	Ad	M	Anal swab, small intestine, spleen	CPV-2a
MT909135	C2018100101	Formosan gem-faced civet	Ad	F	Anal swab, small intestine, spleen	CPV-2a
MT909133	C2018051401	Formosan gem-faced civet	Ad	M	Anal swab, small intestine, spleen	CPV-2a
MT909136	C2018100102	Formosan gem-faced civet	Ad	F	Small intestine, spleen	CPV-2a
MT909143	C2017050902	Ferret-badger	Ad	M	Anal swab	CPV-2a
MT909138	C2018060602	Formosan gem-faced civet	Ad	M	Anal swab, small intestine, spleen	CPV-2b
MN445578	2018060101	Ferret-badger	Ad	F	Anal swab	CPV-2c
MN445583	2017041601	Crab-Eating Mongoose	Ad	M	Anal swab	CPV-2c
MN445584	2017041401	Formosan gem-faced civet	U[1]	U[1]	EDTA, Anal swab	CPV-2c
MT909139	C2016021701	Formosan gem-faced civet	U[1]	U[1]	Small intestine, spleen	CPV-2c
MT909140	C2017032601	Ferret-badger	U[1]	F	Anal swab	CPV-2c
MT909141	C2017041501	Formosan gem-faced civet	Ad	F	Anal swab, small intestine, spleen	CPV-2c
MT909142	C2017041701	Ferret-badger	Ad	F	Anal swab	CPV-2c
MT909144	C2018051401	Formosan gem-faced civet	Ad	M	Anal swab, small intestine, spleen	CPV-2c
MT909146	C2019041801	Formosan gem-faced civet	Juv	M	Small intestine, spleen	CPV-2c
MT909147	C2018070401	Formosan gem-faced civet	Ad	F	Small intestine, spleen	CPV-2c
MT909148	C2019102201	Ferret-badger	Ad	F	Anal swab	CPV-2c
MN445587	2016010101	Crab-Eating Mongoose	Juv	F	EDTA	CPV-2c
MT909145	C2016012701	Crab-Eating Mongoose	Ad	U1	Anal swab, small intestine	CPV-2c
MT909127	C2017061201	Formosan gem-faced civet	Ad	M	Spleen	FPV
MN445586	2016122101	Crab-Eating Mongoose	Juv	F	Anal swab	FPV
MN445588	2015120101	Crab-Eating Mongoose	Juv	M	Anal swab	FPV
MN445589	2015021102	Ferret-badger	Ad	M	Anal swab	FPV

[1] U = unknown.
